# LytR-CpsA-Psr Glycopolymer Transferases: Essential Bricks in Gram-Positive Bacterial Cell Wall Assembly

**DOI:** 10.3390/ijms22020908

**Published:** 2021-01-18

**Authors:** Cordula Stefanović, Fiona F. Hager, Christina Schäffer

**Affiliations:** Department of NanoBiotechnology, NanoGlycobiology Unit, Universität für Bodenkultur Wien, Muthgasse 11, A-1190 Vienna, Austria; cordula.stefanovic@boku.ac.at (C.S.); fiona.hager@boku.ac.at (F.F.H.)

**Keywords:** gram-positive bacteria, cell wall glycopolymers, peptidoglycan modification, glycopolymer ligation, carbohydrate-active enzyme, antibacterial target

## Abstract

The cell walls of Gram-positive bacteria contain a variety of glycopolymers (CWGPs), a significant proportion of which are covalently linked to the peptidoglycan (PGN) scaffolding structure. Prominent CWGPs include wall teichoic acids of *Staphylococcus aureus*, streptococcal capsules, mycobacterial arabinogalactan, and rhamnose-containing polysaccharides of lactic acid bacteria. CWGPs serve important roles in bacterial cellular functions, morphology, and virulence. Despite evident differences in composition, structure and underlaying biosynthesis pathways, the final ligation step of CWGPs to the PGN backbone involves a conserved class of enzymes—the LytR-CpsA-Psr (LCP) transferases. Typically, the enzymes are present in multiple copies displaying partly functional redundancy and/or preference for a distinct CWGP type. LCP enzymes require a lipid-phosphate-linked glycan precursor substrate and catalyse, with a certain degree of promiscuity, CWGP transfer to PGN of different maturation stages, according to in vitro evidence. The prototype attachment mode is that to the C6-OH of *N*-acetylmuramic acid residues via installation of a phosphodiester bond. In some cases, attachment proceeds to *N*-acetylglucosamine residues of PGN—in the case of the *Streptococcus agalactiae* capsule, even without involvement of a phosphate bond. A novel aspect of LCP enzymes concerns a predicted role in protein glycosylation in *Actinomyces oris*. Available crystal structures provide further insight into the catalytic mechanism of this biologically important class of enzymes, which are gaining attention as new targets for antibacterial drug discovery to counteract the emergence of multidrug resistant bacteria.

## 1. Introduction to the Review

This review covers what is currently known about the functions and structures of the LytR-CpsA-Psr (LCP) class of enzymes, which commonly transfer the reducing end of cell wall glycopolymers (CWGPs) of Gram-positive bacteria from a lipid carrier-bound CWGP intermediate to the peptidoglycan (PGN) backbone, usually via a phosphodiester linkage. The review starts with a brief description of the different classes of CWGPs and the LCP enzymes themselves before going on to a species-by-species discussion. Both molecular and structural biology of the systems that have so far been studied are presented as well as the questions that remain to be addressed for the future evaluation of LCP enzymes as novel antibacterial targets.

## 2. Cell Wall Glycopolymers—Brief Insight into Composition and Biosynthesis

### 2.1. Peptidoglycan (PGN)—A General Perspective

In Gram-positive bacteria, the cytoplasmic membrane is surrounded by a remarkably dynamic and constantly remodelled thick layer of the bacterial cell wall material peptidoglycan (PGN) [1,2]. PGN is a complex macromolecule (“PGN sacculus”) that is essential for bacterial survival. It plays an important role against environmental challenges, serves as a protection from rupture due to high internal osmotic pressure, and specifies cell shape [3,4]. PGN is composed of linear glycan backbone strands of alternatingly β-1,4-linked *N*-acetylmuramic acid (MurNAc) and *N*-acetylglucosamine (GlcNAc) residues coupled via stem peptides that are attached to the MurNAc residues, resulting in a crosslinked, mesh-like framework. These cross-linkages via different stem peptides together with possible interpeptide bridges, such as the well-known pentaglycine bridge in the PGN of *Staphylococcus aureus*, form the basis for the classification of different PGN-types [5]. Importantly, PGN scaffolds numerous proteins and glycopolymers [4,6].

The role of PGN in bacterial survival has made PGN biosynthesis a target for important classes of antibiotics, including the glycopeptides (e.g., vancomycin) and the β-lactams (e.g., the penicillins, cephalosporins, and carbapenems) [7]. Both of these antibiotics inhibit PGN crosslinking, however, in different ways. While vancomycin binds to D-alanyl-D-alanyl groups at the end of the stem peptides of opposing PGN strands, the β-lactams irreversibly bind transpeptidases (penicillin-binding proteins) [8,9,10]. As a mode of induced resistance, bacteria-produced β-lactamases frequently render this group of antibiotics ineffective [11].

Due to the worldwide increase of antibiotic resistances and the lack of novel drugs in the development pipeline, there is a renewed interest in (unexplored) cell wall components and underlaying biosynthesis pathways to find alternate options to treat infections caused by multidrug-resistant bacteria [12]. Furthermore, antibiotic inhibition of bacterial cell wall biosynthesis induces both common and compound-specific transcriptional responses, which both can be exploited to increase antibiotic susceptibility [13].

### 2.2. Types of Glycopolymers Attached to PGN

#### 2.2.1. General Considerations

The covalent attachment of various glycopolymers to PGN (cell wall glycopolymers—CWGP) is an enigmatic feature of the cell wall assembly of Gram-positive bacteria. CWGPs are mostly found to be anionic, typically composed of individual repeating units (RU) of varying degrees of polymerization, and can make up to 50% of the dry weight of the cell wall [14]. Frequently, they are tethered to the C6-OH group of MurNAc residues of the PGN glycan backbone strands via a phosphodiester bond, but also the GlcNAc residues can be modified [4,15,16]. CWGPs are involved in a myriad of crucial cellular functions and serve as a rich source for both validated and unexploited pathways that are essential for bacterial virulence and survival [17,18,19].

On the basis of their structural characteristics, PGN-bound CWGPs of Gram-positive bacteria can be classified into two major groups: (i) “classical” CWGPs, comprising wall teichoic acids (WTAs) [20,21] and teichuronic acids [14,22], and (ii) “non-classical” CWGPs, which is the more diverse group comprising all other anionic and neutral CWGPs [23,24]. To these CWGPs, initially only secondary roles in cell wall function have been attributed; hence, they have been named “secondary” cell wall polymers. However, given the rich body of knowledge that has accumulated over the past 40 years, it is evident that these compounds play pivotal roles in bacterial cell function, physiology and virulence; thus, nowadays, the term “CWGPs” is more appropriate [16].

Based on detailed investigations of the lipopolysacchaide (LPS) biosynthesis in Gram-negative bacteria [25], two major principal assembly routes are known for bacterial CWGPs—the ABC-transporter dependent pathway and the Wzy-dependent pathway [26]. In many bacteria, the genetic information for CWGP biosynthesis is encoded in genomic biosynthesis gene loci or clusters. However, given the interchangeability of modules between the routes, the prediction of a distinct route based on the bacterial genome content is impossible. Generally, the interplay between different CWGP assembly routes—including also that for the PGN scaffold itself—concerns common pools of activated monosaccharides, biosynthetic intermediates, of the C_55_ undecaprenylphosphat (undp-*P*) lipid carrier and, importantly, enzymatic machineries [27]. This becomes evident upon inhibition of individual biosynthetic steps. However, it is still largely unknown how the different CWGP biosynthesis pathways, which share building blocks and membrane carriers, function in a coordinated and integrated fashion [28].

While the coupling of different CWGPs to the PGN has long been discovered [29,30], until the recently exposed LytR-CpsA-Psr (LCP) enzyme family, there were not any plausible candidates for this coupling reaction [29].

In this review, we are discussing different CWGPs from various bacterial sources, for which information on their ligation to PGN is available in the literature. However, studying LCP enzymes is challenging considering that frequently different types of CWGPs are simultaneously present in a bacterium and that the presence of multiple copies of LCP proteins is the rule rather than the exception.

#### 2.2.2. Wall Teichoic Acids

**Figure 1:**
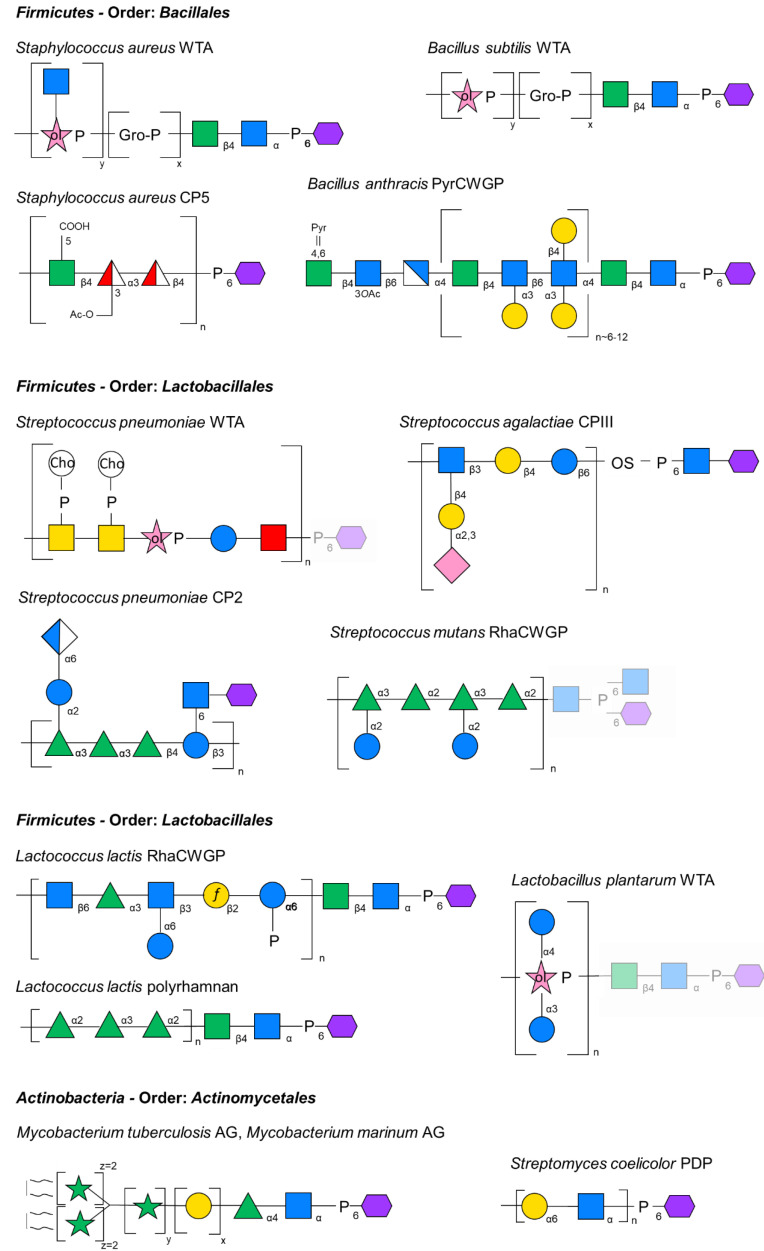
CWGP substrates of LCP proteins. Repeat-unit structures and the (proposed) linkage to PGN are shown, where investigated. Monosaccharide symbols are shown according to the Symbol Nomenclature for Glycans (SNFG) [32].
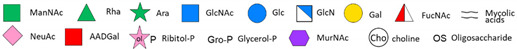

Wall teichoic acids (WTAs) are the most abundant and best-investigated PGN-linked CWGPs in many Gram-positive organisms [14]. They are composed of repeating units of alditol phosphates linked by phosphodiester bonds and can be further substituted by amino acids (e.g., D-alanine) and/or carbohydrate residues. Typically, *Bacillus subtilis* produces glycerol phosphate (Gro-*P*) or ribitol phosphate (Rbo-*P*) WTAs, while in *S. aureus*, commonly Rbo-*P* WTAs are present [16,31] (Figure 1).

In either case, a conserved β-D-Man*p*NAc-(1→4)-α-D-Glc*p*NAc-(1→P murein-linkage unit at the reducing end of the glycopolymer mediates WTA attachment to the PGN. In fact, this “conventional” murein linkage unit is also found in several other CWGPs, as is explained below.

The assembly route of WTAs follows the ABC-transporter dependent route [18,19]. This implicates CWGP assembly on a C_55_ undp-*P* carrier in the cytoplasm, involving the formation of the “conventional” β-D-Man*p*NAc-(1→4)-α-D-Glc*p*NAc-(1→*P* murein linkage unit and polymer elongation using individual nucleoside-diphosphate (NDP)-bound substrates in concert with dedicated glycosyltransferases, followed by glycopolymer export to the outer leaflet of the membrane by an ABC-transporter. Upon reaching the exterior of the cell, WTAs are further functionalized and, finally, transferred to PGN in a ligation reaction involving LCP enzymes [19]. Conserved enzymes for the biosynthesis of the “conventional” murein linkage unit are TagO and TagA [18,33,34]. The first enzyme in this pathway, TagO, is an integral membrane protein that transfers GlcNAc-phosphate from UDP-GlcNAc to the undp-*P* lipid embedded in the cytoplasmic membrane [35,36]. The lipid-linked monosaccharide is then elongated to a disaccharide by the UDP-ManNAc transferase TagA [37,38,39]. This lipid-linked disaccharide constitutes the platform for the subsequent steps of WTA biosynthesis [29,40,41,42,43] and other CWGPs [44,45].

WTAs are intimately involved in many aspects of cell division and essential for maintaining cell shape in rod-shaped organisms [41]. Importantly, WTAs are key determinants of virulence and antibiotic resistance—e.g., in methicillin-resistant staphylococci (MRSA) or in streptococci—and have, thus, been the target of numerous screening campaigns to find inhibitors [46,47].

#### 2.2.3. Pyruvylated CWGPs

Pyruvylated CWGPs (PyrCWGP) are a less investigated class of PGN-attached CWGPs which are of interest in the context of protein cell surface display in Gram-positive bacteria [48]. These SCWPs are 5–20 kDa in size, composed of species-specific repeats [6,49], but lack repetitive alditol phosphates and phosphodiester bonds of WTAs [15,50,51], hence, categorized as “non-classical” CWGPs. Importantly, they contain 4,6-pyruvateketal-modified-β-D-*N*-acetylmannosamine (Pyr-β-D-Man*p*NAc) imparting a negative charge, which serves as a specific cell wall ligand for S-layer homology (SLH) domains that are usually present in triplicates at the termini of cell surface proteins [48,52,53]. Prominent examples of SLH-domain proteins are surface (S-) layer proteins which self-assemble into two-dimensional crystalline arrays on the bacterial cell surface [54,55]. S-layer proteins are important for many biological functions such as maintenance of cell integrity, enzyme display, protection to phagocytosis, and interactions with the host and its immune system [56].

Best investigated pyruvylated CWGPs are those from *Paenibacillus alvei* CCM 2051^T^ and *Bacillus anthracis*. The *B. anthracis* CWGP is composed of →4)-β-ManNAc-(1→4)-β-GlcNAc-(1→6)-α-GlcNAc-(1→ trisaccharide RUs with strain-dependent galactosylation occurring at the GlcNAc residues [57,58]; Pyr-β-D-Man*p*NAc is exclusively contained in the terminal repeat [51,57,58,59] (Figure 1). In contrast, the CWGP of *P. alvei* is multiply pyruvylated and consists of →3)-Pyr-β-D-Man*p*NAc-(1→4)-β-D-Glc*p*NAc-(1→ RUs [60,61].

Notably, a common structural feature of WTAs and pyruvylated CWGPs is the presence of the “conventional” β-D-Man*p*NAc-(1→4)-α-D-Glc*p*NAc-(1→*P* murein linkage unit.

#### 2.2.4. Capsules

Capsules are long-chain polysaccharides (CPS), which are produced by many bacteria of both Gram-classes [62]. They can either maintain a strong association with the enveloping bacterial cell and/or be secreted into the immediate environment in the form of exopolysaccharides [63]. Capsules afford the producing bacteria protection from a wide range of physical, chemical, and biological stresses, support biofilms, and play critical roles in interactions between bacteria and their immediate environments [63].

The Wzy pathway constitutes a prototypical mechanism to produce these structures. Briefly, the basic CPS RUs are synthesized at the cytosolic side of the membrane by the sequential action of glycosyltransferases using NDP-sugar substrates. The RU is anchored to a undp-*P* membrane lipid, and it is transferred to the outer side of the membrane via a Wzx flippase, where it is polymerized into the full-length CPS by the addition of new repeat units to the reducing end of the glycopolymer in a reaction requiring the Wzy polymerase and the chain-length regulator Wzz. In some Gram-positive bacteria, the lipid-bound CPS precursors serve as substrates for LCP enzyme-catalysed coupling of CPS to PGN, thus creating the mucoid capsule layer covering the bacterial surface [28,63,64].

#### 2.2.5. Arabinogalactan

Arabinogalactan (AG) is a heteropolysaccharide found in covalent attachment to PGN via a phosphoryl-*N*-acetylglucosaminyl-rhamnosyl linkage unit with the structure →4)-α-L-Rha*p*-(1→3)-α-D-Glc*p*NAc-(1→*P* to MurNAc residues of the mycobacterial A1γ-type PGN (Figure 1). AG comprises a galactofuran domain bound to the linkage unit which is extended by the arabinofuran domain, which is, in turn, esterified at its non-reducing ends to long-chain (C_70_–C_90_) mycolic acids forming the inner leaflet of the mycomembrane [65,66].

Notably, mycobacteria stain Gram-indifferently and the compositional and architectural complexity of the mycobacterial cell envelope distinguishes species of the *Mycobacterium* genus from other prokaryotes. It is the basis of many of the physiological and pathogenic features of mycobacteria and the site of susceptibility and resistance to many anti-tuberculosis drugs [67,68].

The synthesis of AG is initiated in the cytoplasm on a C_50_-undp-*P* carrier lipid with formation of the murein linkage unit [69,70] followed by the addition of Gal*f* and Ara*f* residues [71,72,73] and AG export, potentially involving an ABC-transporter [74]. It is thought that the GlcNAc residue of the linkage unit of the mature AG next forms a 1-*O*-phosphoryl linkage with the C6-OH position of a MurNAc residue of PGN [71] and that this transfer reaction is catalysed by an LCP family protein [65], which requires newly synthesized PGN undergoing concomitant cross-linking [75].

Despite the fundamental structural differences that exist between AG and WTAs, the structure of the AG-PGN linker shares similarity with the “conventional” murein linkage unit of WTAs with regard to the reducing-end GlcNAc residue and, evidently, LCP proteins are involved in the final ligation of AG to PGN [66].

#### 2.2.6. Rhamnose-Containing Cell Wall Glycopolymers

Several members of the order *Lactobacillales* (mainly streptococcal species and *Lactococcus lactis*) can generate a specific class of abundant CWGPs, which are composed of individual RUs, where rhamnose is the major constituent, along with variable combinations and linkages of Glc, GlcNAc, Gal, GalNAc, and phosphate [76]. This heterogeneous group of rhamnose-containing CWGPs is named RhaCWGPs [76].

For the Group B *Streptococcus* carbohydrate (GBC) and Group G carbohydrate (GGC), rhamnose is the major antigenic determinant. Species carrying these structures are serologically discriminated based on the presence of either a single rhamnose in GGC versus triterminal α-L-(1→2)-Rha*p* in GBC in the RhaCWGP RU (for review, see [76]). Among the streptococcal group antigens, the GBC is unique since it forms a multiantenna branching structure and is negatively charged, due to the presence of phosphodiester bonds that link different GBC repeat units. Similarly, the *L. lactis* RhaCWGP contains phosphodiester bonds that link branched →6)-β-GlcNAc-(1→3)-Rha-(1→3)[α-Glc–(1→6)]-β-GlcNAc-(1→2)-β-Gal*f*-(1→6)-α-Glc-*P*-(1→ hexasaccharide RUs [77] (Figure 1).

RhaCWGPs comprise about 40–60% of the bacterial cell wall by weight, and they are localized on the outermost surface of the cell wall but are likely also intercalated within the PGN layer, since antibodies directed against these structures bind to both sides of isolated cell walls [78]. According to recent evidence obtained for the glucose-containing RhaCWGP of *Streptococcus mutans* (also referred to as rhamnose-glucose polysaccharide–RGP) (Figure 1), RhaCWGPs are assembled on a lipid carrier in the cytoplasm and exported via an ABC-transporter followed by covalent attachment to the PGN via the likely activity of an LCP family protein [79].

Of note, L-rhamnose as integral constituent of RhaCWGPs is often essential for bacterial virulence or even viability [80], making its biosynthesis pathway an attractive therapeutic target.

## 3. Lytr-CpsA-Psr (LCP) Enzymes—A General Perspective

The LCP family of proteins is a conserved family of phosphotransferases catalysing the formation of a phosphodiester bond to link CWGPs onto the MurNAc or GlcNAc residues of PGN. These enzymes have aroused great interest because of their role in Gram-positive bacterial cell envelope maintenance and influence on various virulence factors as well as antibiotic resistance of human pathogens [40,49,81]. Members of this enzyme family were discovered in eight bacterial phyla—*Actinobacteria, Bacteroidetes*, *Chloroflexi*, *Cyanobacteria*, *Deinococcus-Thermus*, *Firmicutes*, *Spirochaetes*, and *Thermotogae*, and their Lytr-CpsA-Psr (LCP) domain is unique to the bacterial kingdom [49]. The “LCP” acronym derives from three proteins initially identified to contain a LytR domain—LytR (lytic repressor, now TagU5), CpsA (capsular polysaccharide expression regulator), and Psr (PBP 5 synthesis repressor).

Typically, LCP proteins have a common structural organization made up of an N-terminal transmembrane (TM) domain required for anchoring, an optional, non-conserved accessory domain (CATH 3tflA01), a core catalytic domain that is predicted to be extracellular, and, sometimes, a C-terminal domain of unknown structure. Furthermore, the core LCP domain is a magnesium-dependent enzyme [82].

**Figure 2:**
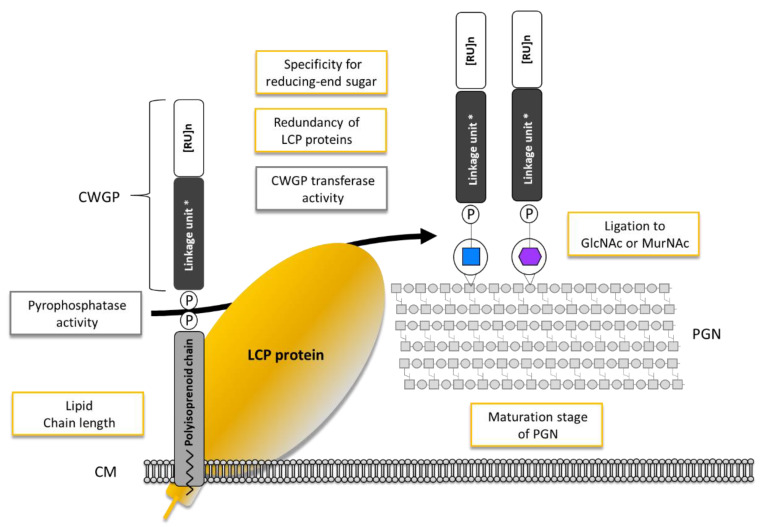
Most common scenarios of ligation of CWGPs to PGN by LCP enzyme activity. The reaction is shown with a schematic CWGP structure comprised of the glycopolymer chain and a possible PGN linkage unit (*). LCP protein functions are boxed in black, open key points of the catalytic reaction are boxed in yellow. CM, cytoplasmic membrane; P, phosphate.

Despite their great abundance in Gram-positive bacteria, the precise role of LCP enzymes in cell wall assembly and their catalytic function(s) are only beginning to be discovered. There is evidence that LCP enzymes utilize C_55_ lipid-phosphate bound CWGP precursor substrates and that the ligation process likely releases the lipid carrier, which enters new synthesis cycles. Several cardinal points of LCP enzyme activity are remaining to be clarified in future studies. From a catalytic perspective, these concern i) enzyme stringency versus promiscuity for the glycopolymer’s PGN linkage unit, ii) relevance of the polyisoprenoid portion of the CWGP precursor, and iii) role of the maturation stage of the PGN acceptor. From a biological perspective, points to be clarified concern iv) the role of multiple LCP proteins in a given bacterium, i.e., if there is (partly) functional redundancy or preference for the transfer of a distinct of coexisting CWGPs, and v) the intracellular control for LCP enzyme expression (Figure 2).

## 4. LCP Enzymes According to Bacterial Species

Experimental evidence of LCP enzyme function is available for bacteria affiliated to the phyla *Firmicutes*, *Actinobacteria*, and *Cyanobacteria*.

### 4.1. Firmicutes—*Order*: Bacillales

#### 4.1.1. *Staphylococcus aureus*

*S. aureus* is a Gram-positive opportunistic pathogen of which certain strains have become resistant to most antibiotic classes [83,84,85]. The bacterium can lead to systemic failures, such as infective endocarditis or bacteraemia via nosocomial acquisition [83,86].

The *S. aureus* WTA is a polymer of 30 to 50 Rbo-*P* subunits connected via 1,5-phosphodiester bonds [16], which is tethered to PGN via the “conventional” murein linkage unit [87] (Figure 1). Furthermore, *S. aureus* expresses a CPS, which substantially contributes to the bacterium’s ability to cause invasive disease [88]. Specifically, capsular polysaccharide type 5 (CP5) is composed of →4)-β-D-Man*p*NAcA-(1→3)-α-L-Fuc*p*NAc-(1→4)-β-D-Fuc*p*NAc-(1→ repeats with *O*-acetylation on all L-Fuc*p*NAc residues except for the one in the reducing-end RU [28] (Figure 1). Of note, CP5 biosynthesis is TagO-independent resulting in the absence of a “conventional” murein linkage unit in the capsule.

##### *S. aureus* LcpA, LcpB, LcpC at a Glance

The *S. aureus* genome harbours three LCP proteins, encoded by *lcpA* (previous name, *msrR*), *lcpB* (*sa0908*), and *lcpC* (*sa2103*) [49,89], which are in part functionally redundant regarding cellular functions [29,90].

*S. aureus* variants carrying defective alleles of *lcp* genes resulted in enhanced [91,92] susceptibility to β-lactam antibiotics, deviant septum formation [91,92], autolysis [91], activation of a cell wall stress response [93], reduced phosphate content of staphylococcal cell walls [93], and aberrant biofilm formation [92].

In strains lacking WTA due to the inactivation of LCP function, major cell division defects were shown, the PGN synthesis machinery was not localized properly, and these strains were unable of nasal epithelial cell colonization [94,95]. Furthermore, methicillin-resistant *S. aureus* (MRSA) strains were found to become sensitive to β-lactam antibiotics when WTA synthesis was abrogated [95,96].

##### *S. aureus* LcpA, LcpB, LcpC in CWGP Biosynthesis

Due to their demonstrated biological importance, the biochemical activity of *S. aureus* LCP proteins is under intense investigation [89].

Deletion of all three LCP genes resulted in complete WTA loss in the staphylococcal cell wall and deletion of any of individual LCP genes disturbed the attachment of WTA in different degrees [89]. This partial functional redundancy was also seen for different phenotypes including β-lactam resistance, biofilm formation, and growth defects [91]. Of note, LcpA was shown to be the most important protein related to WTA-functions [84].

In a reconstitution approach, cognate Rbo-*P* WTA precursors including the murein-linkage unit (Figure 1) could be transferred to PGN by truncated versions of either of the three staphylococcal LCP proteins devoid of the TM-segment (ΔTM) [29], without the requirement of any other proteins, as had been initially suggested [82]. For the ligation, the WTA substrate needs to be comprised of only two sugar residues (i.e., the “conventional” murein linkage unit) and a hexaprenyl chain, mimicking the truncated native C_55_ undp lipid. This is consistent with the length of the hydrophobic channel length observed in the crystal structure of Cps2A—the LCP protein transferring CPS in *Sc. pneumoniae* [82,97]. Importantly, the *S. aureus* WTA precursor could be transferred to “nascent” (i.e., un-crosslinked) PGN polymers only [98], not to lipid II (*i.e*., murein pentapeptides) [29], indicating that modification of PGN with WTA occurs prior to final PGN cross-linking [98]. Deletion of *lcpC* had no effect on the level of WTA that was ligated to PGN, whereas a Δ*lcpA* or a Δ*lcpB* mutant showed reduced levels of WTA content [89,93]. This is corroborated by the finding that the Δ*lcpC* mutant showed no phosphate release compared to single Δ*lcpA* and Δ*lcpB* mutants [89]. Differential localization and regulation of the enzymes might be important factors regarding the greater impact of LcpA and LcpB on WTA synthesis [93].

Genes involved in *S. aureus* CPS synthesis, exemplified with CP5 (Figure 1), are clustered presenting conserved genes employing a Wzy-dependent biosynthesis mechanism [64,90], and there is evidence that specifically LcpC plays a key role in catalysis of *S. aureus* capsule attachment to the PGN [28,29,90]. This is corroborated by the lack of a *cpsA* homologue encoding a CPS ligase, typically involved in streptococcal CPS ligation, in the *S. aureus* genome [49,99]. Initial evidence of the requirement of LcpC for ligation of *S. aureus* CP5 to PGN was derived from a mutant approach [90]. The Δ*lcpC* mutant accumulated the capsule in the supernatant fraction, while the Δ*lcpAB* variants did not display any defect in CP5 synthesis [90]. A triple mutant showed the same levels of CP5 reduction as the variant lacking *lcpC.* Surprisingly, plasmid-based expression of any of the three *lcp* genes could restore the CP5 content, indicating that all three LCP proteins are to some extent involved in the attachment [90].

To investigate the proposed role of LcpC in vitro, different [^14^C] CP5 lipid intermediates were synthesized, including lipid I_cap_ (i.e., C_55_ undp-*PP*-D-FucNAc), lipid II_cap_ (i.e., C_55_ undp-*PP*-D-FucNAc-L-FucNAc), and lipid III_cap_ (i.e., C_55_ undp-*PP*-D-FucNAc-L-FucNAc-D-ManNAcA). After purification, these were used together with the ultimate PGN precursor lipid II (lipid II_PGN_), i.e., C_55_ undp-*PP*-D-MurNAc-D-GlcNAc including a pentapeptide, as a potential acceptor substrate [28]. In this setup, LcpC was able to catalyse cleavage of the donor substrate lipid I_cap_ and catalyse attachment of the phosphoryl-sugar moiety to the ultimate PGN precursor lipid II. Strikingly, in the presence of CapA1, an activator/phosphodiesterase protein that cleaves lipid-PP-linked CP5 precursors [28], the transfer rate was increased, possibly by forming an interaction complex between CapA1 and LcpC [28,100]. Surprisingly, in the case of lipid II_cap_, no transfer to the PGN acceptor could be overserved in the LcpC in vitro assay, which would likely be deleterious in vivo. On the other hand, all CP5 lipid intermediates were effectively processed by LcpC, although the proximal undp-*PP*-linked FucNAc residue appeared to be sufficient for CP5 precursor recognition. Of note, the natural PGN acceptor substrate of LcpC remains elusive; possible acceptor structures include lipid II_PGN_, as well as “nascent” and cross-linked PGN.

Summarizing, there is evidence that LcpC preferentially recognizes CP5 intermediates rather than WTA intermediates where deletion of *lcpC* caused only minor reduction levels [89,90]. Whether this is due to the different reducing-end sugars (i.e., D-FucNAc in the CP5 repeat versus D-Glc*p*NAc in the “conventional” WTA murein linkage unit remains to be investigated. Notably, in LPS O-antigen RU biosynthesis, the first C_55_ lipid-*PP*-linked sugar unit of the O-antigen RU contains all necessary recognition information for the catalytic activity of the O-antigen ligase WaaL [101].

##### *S. aureus* lcpA, lcpB, lcpC Genes and Physiological Effects

Deletion of individual *S. aureus* LCP protein encoding genes showed only minor effects on *S. aureus* cell wall physiology and growth, whereas a Δ*lcp* triple mutant was barely viable, showing temperature sensitivity and enlarged cells [91]. Complementation with LcpA resulted in restoration of growth and cell size almost to wild-type size [91].

Interestingly, Δ*lcp* and Δ*lcp* Δ*tagO* variants abolished cell division planes by generating aberrant cells with irregular envelopes lacking WTA [89]. Of note, deletion of *tagO* alone did not abolish staphylococcal growth [35,39,102]. The same phenotype was observed for isolated *tagO* and *lcpA* mutants; however, deletion of *lcpA* did not interrupt WTA synthesis and thus suggests that WTA synthesis as well as assembly are crucial for normal cell division [89,95]. Furthermore, inhibition of WTA synthesis by tunicamycin treatment did not relieve deviant cell separation of the mutant cells and, therefore, did not suppress the cell division defects of Δ*lcp* variants [89].

Autolysis was induced by deletion of all three LCP proteins, resulting in an increased resistance of the Δ*lcp* triple mutant to autolysis compared to the wild-type [89]. Complementation by LcpA increased autolysis levels the most [89]. These pleiotropic phenotypes in the Δ*lcp* mutant are likely owed to WTA cell wall deposition defects and WTA synthesis, as staphylococcal cells without WTA resulted in deviant cell size and septum formation as well as susceptibility to antibiotics and biofilm formation defects [49,91,92,93].

A comparison of the surface proteomes of methicillin-resistant, laboratory-adapted *S. aureus* COL strain (COL) and a COL strain in vitro-adapted to high levels of oxacillin (APT) was used to characterize virulence factors showing that LcpC was found uniquely on the APT surface, suggesting a role in adaption to high oxacillin levels [84]. Deletion of *lcpA* decreased oxacillin resistance and upregulated *lcpA* expression was observed by triggered antibiotic stress [103] and, furthermore, increased cell size and elevated cell wall remodelling was revealed [84,92]. However, overexpression of LcpC did not generate the APT phenotype in COL, suggesting that aggregation and changes in cell morphology are multifactorial [84]. The adaption of LcpC to high levels of oxacillin in the APT strain prompted the question about the contribution of this enzyme to antimicrobial resistance and pathogenicity [104]. The finding that deletion of *lcpC* decreased the resistance to β-lactams in methicillin-resistant *S. aureus* (MRSA) and in methicillin-susceptible *S. aureus* (MSSA) and, consequently, the pathogenicity in the host, suggested that the deviant cell shape might allow for an easier access of the antibiotics to the cell [104]. Thus, LcpC could be an effective target for drug development [104].

In MSSA and MRSA strains, reduced oxacillin resistance levels were also observed upon *lcpA* deletion [91,92,103]. The Δ*lcp* triple mutant was hypersusceptible to oxacillin and growth could be restored by any of the three LCP proteins; LcpA had the greatest impact, followed by LcpB and LcpC [91]. Interestingly, the Δ*lcpA* mutant produced more biofilm, and in the Δ*lcp* triple mutant complementation with LcpC revealed the strongest biofilm, LcpA the weakest [91]. A mutation in *lcpA* (E146K) was shown to have an impact on β-lactam and vancomycin resistances and led to a reduced resistance to oxacillin, whereby the cells showed abnormal septal placement [105]. The mutation further led to a decreased autolytic activity, as was also evident form highest autolysis levels obtained after complementation of the Δ*lcp* triple mutant with LcpA [91,105].

##### Crystal Structure of *S. aureus* LcpA

**Figure 3:**
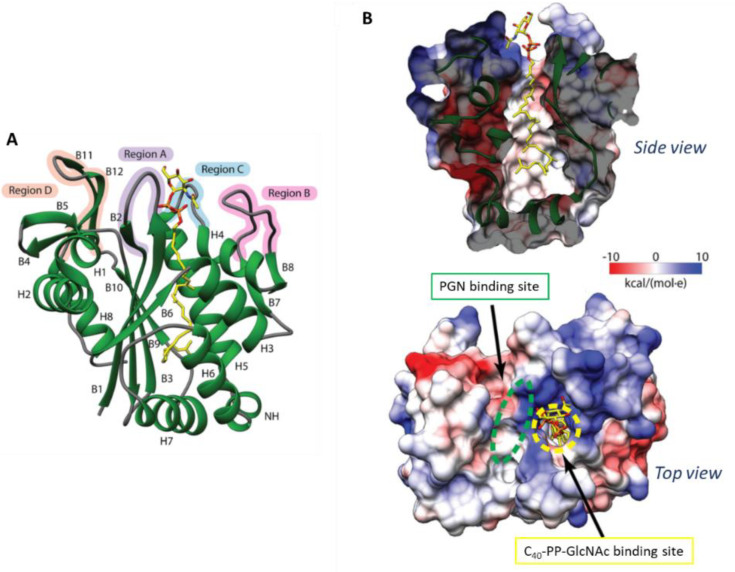
Crystal structure of *S. aureus* LcpA. (**A**) ribbon structure of LcpA (green) bound to C_40_-*PP*–GlcNAc (yellow) with labelled structural features. H, helix; B, β-strand; NH, non-conserved helix. The active site is surrounded by four regions, designated here as A, B, C, and D. (**B**) The lipid-binding pocket of LcpA is shown in a cross-section of a side view with an electrostatic potential surface. The locations of the substrate-binding sites are outlined in the top view, figure modified after [106].

The crystal structure of LcpA devoid of its TM (residues 80–327; ΔTM-LcpA) complexed to C_40_-*PP*-GlcNAc was solved to 1.9 Å and provides a relevant target for inhibitor design studies [106] (Figure 3). The extracellular domain consists of six-stranded β-sheets overlaid amongst several α-helices and double-stranded β-sheets, where a hydrophobic binding pocket narrow opening and a wide base is formed from its center [106]. The active site of LcpA is enclosed by region A (residues 92–100), B (residues 188–201), C (residues 217–224) and D (residues 296–312) and shows structural variability. The electropositive region in the active site, as seen in other LCP enzymes, consists of conserved arginine residues. R218 located in the loop of the highly flexible region C is suggested to aid in product expulsion and is held away from the active site by a salt bridge. Furthermore, three potential PGN saccharide binding sites were identified in close proximity to the conserved regions of R99, K135, N137, and D224. As D123 is conserved in many LCP enzymes, it is likely that this basic amino acid plays an important role in PGN binding [106].

#### 4.1.2. *Bacillus subtilis*

WTAs and lipoteichoic acid constitute up to 60% of the dry weight of the cell wall in *B. subtilis* providing an overall negative charge to the cell wall [14,107]. Both WTA and LTA are important, as cells that cannot produce either of these compounds show morphological aberrations and can only be grown under certain conditions, whereas the absence of both CWGPs is lethal [41]. WTA is covalently attached to the MurNAc residues in the *B. subtilis* cell wall via a “conventional” murein linkage unit; coupled to it is poly(Gro-*P*) that can have either D-alanine or glucose bound to the C2, with chain lengths varying from 45 to 60 residues [14] (Figure 1).

WTA biosynthesis in *B. subtilis* has traditionally been intensely investigated [14,108].

##### *B. subtilis* TagT, TagU, TagV, Genes and Physiological Effects

In the *B. subtilis* genome, three LCP genes are encoded—*tagT* (previously named *ywtF*), *tagU* (*lytR*), and *tagV* (*yvhJ*) [82]. As in *S. aureus*, also in *B. subtilis*, *lcp* gene deletion mutants revealed defects in WTA, accompanied by reduced virulence, enhanced antibiotics susceptibility, and deviant cell wall structures [82,91,92]. Single mutation variants of the *tagTUV* genes showed no significant effect on either cell morphology or growth, but a Δ*tagTV* mutant displayed noticeably slower growth and aberrant cell shape [82].

In their search for interaction partners for the MreB protein that is involved in later wall PGN synthesis in *B. subtilis*, Kawai et al. were among the first to describe the LCP enzyme family and identified these enzymes as key players in the attachment of anionic CWGPs such as WTA to the cell wall [82]. The expression of at least one out of the three *lcp* gene was found to be required for complete WTA biosynthesis and growth of *B. subtilis* [82].

##### *B. subtilis* TagT, TagU, TagV in WTA Biosynthesis

It was demonstrated in in vitro assays that all LCP enzymes, produced recombinantly without the TM domain, were capable of transferring WTA intermediates to PGN [109]. Particularly, lipid β (i.e., Man*p*NAc-β-(1→4)-Glc*p*NAc-1-*PP*-undp) was attached to mature PGN in vitro [109], which is consistent with several crystal structures of LCP enzymes where these proteins showed to bind polyisoprenoid phosphate lipids [82,109]. Of note, the use of a short aliphatic chain (C_13_) in place of the authentic C_55_ undp moiety led to a 95% reduction of LCP activity [109]. This contradicts the observations made by others for *S. aureus* LCP proteins, where the lipid portion was assumed to play only a minor role in the ligation reaction [82].

Currently, it is suggested that key interactions between LCP enzymes and donor CWGP substrates occur in the region of the polyisoprenoid lipid that is proximal to the pyrophosphate moiety. Interestingly, the individual *B. subtilis* LCP enzymes display significant differences in their in vitro activities [109]. TagU showed an approximately three-fold higher activity compared to TagT and TagV, which contradicts the proposed functional redundancy of the proteins [82,109]. Thus, it is still possible that each Tag enzyme transfers preferentially a distinct type of CWGP to PGN, representing a scenario comparable to *S. aureus*, where LcpA [98] and LcpC [90] can recognize different substrates [109].

Conclusively, catalysis of WTA transfer by *B. subtilis* LCP enzymes requires magnesium ions and a polyisoprenoid moiety of the donor CWGP [109], and this finding is in accordance with the identity of the first three isoprene units of a WTA substrate used by Schaefer et al. [29,109]. Notably, WTA transfer by TagT, TagU and TagV was successful to higher-order structures of PGN, but it still needs to be investigated how the oligomeric repeats of PGN are recognized.

##### Crystal Structure of *B. subtilis* TagT, TagU, TagV

A ΔTM-TagT (residues 44-322) construct was crystallized, and, consistent with its known pyrophosphatase activity towards polyprenoid-pyrophosphate lipid substrates [82], an octaprenyl-pyrophosphate fitted well in the hydrophobic tunnel in the electron density map [97]. Interestingly, longer lipids, such as undp-*P* fitted worse, and presumably in a full-length protein, the hydrophobic tail of the lipid extends out of the tunnel to interact with the membrane. The ΔTM-TagT structure supports the enzymatic role of TagT in the activity of transferring phosphorylated anionic CWGPs from an undp-*P*-linked precursor to PGN [97].

Many interactions between the pyrophosphate head group and charged residues are similar to the *Sc. pneumoniae* Cps2A enzyme (see below). Asp82, corresponding to Asp234 in Cps2A, is more distant from the phosphate, whereas Asp234 coordinates a magnesium ion interacting with the lipid head group. However, this loop region showed disorder and could not be modelled and possibly is the reason why the magnesium ion did not bind to the pyrophosphate group [97].

In a more recent study to better understand the substrate preferences of LCP proteins, the ΔTM-TagT protein was crystallized with two lipid-linked WTA precursors, namely LI^WTA^ (i.e., [^14^C] lipid-*PP*-GlcNAc) and LII^WTA^, containing the “conventional” disaccharide murein linkage unit [98]. The resulting structures differed regarding the orientation of the pyrophosphate and saccharide moieties, where the disaccharide-containing structure exposed a divalent cation in the active site. Changes in the glycosidic linkage and pyrophosphate bonds led to a different orientation of the GlcNAc residue of LII^WTA^, resulting in a conformation of the pyrophosphate where a divalent cation can bind, as would be necessary for catalysis. Evidently, the PGN substrate binds closely to the anomeric phosphate of LII^WTA^ in a narrow groove, which, similar to the WTA binding pocket, displays three conserved arginine residues. R219 is suggested to play a role in the nucleophilic attack by the PGN substrate, whereas another arginine residue, R118, facilitates deprotonation of the C6-OH of MurNAc [98].

Li and colleagues provided crystal structures of all three *B. subtilis* LCP proteins, with additional electron density for the disordered loop region in the TagT apo structure. All enzymes were expressed without their TM segment (TagT, residues 46–322; TagU, residues 62–306; TagV, residues 72–332). All secondary structures consist of four regions, consistent with the structure of LcpA from *S. aureus* (compare with Figure 3), with significant differences in region B, where TagT shows an α-helix, and TagU and TagV a double-stranded β-sheet. On one side of the central β-sheet in TagU, helices 3–7 collapse the lipid binding site, whereas other LCP enzymes, including TagT, show a disorder of helix 6. The exposure of this hydrophobic core is, again, suggested to be necessary for surface interaction with the membrane [106].

#### 4.1.3. *Bacillus anthracis*

*B. anthracis* is a spore-forming Gram-positive pathogen which replicates within vertebrates as chains of vegetative cells by regulating the separation of septal PGN [110]. The pathogen is the causative agent of anthrax and displays a unique growth pattern by tethering at septal PGN, which results in protection of the bacterium from engulfment by host phagocytes [111].

Comparing *B. anthracis* with *S. aureus* and *B. subtilis* with regard to the presence of CWGPs, it is important to note that *B. anthracis* does not harbour a WTA, but expresses a PyrCWGP [48] (Figure 1) and a poly-D-γ-glutamic acid capsule that is bound to PGN via amide bonds [112,113]. The *B. anthracis* S-layer proteins and S-layer-associated proteins (BSLs) [114] function as chain length and cell size determinants and are assembled in the envelope by binding to the bacterium’s PyrCWGP [48]. The biosynthesis of that specific CWGP involves the *B. anthracis* LCP proteins [43,115,116].

##### *B. anthracis* LcpB1, LcpB2, LcpB3, LcpB4, LcpC, LcpD, Genes and Physiological Effects

*B. anthracis* encodes on its genome six LCP homologues—BAS1830 (LcpB1), BAS0572 (LcpB2), BAS0746 (LcpB3), BAS3381 (LcpB4), BAS5115 (LcpC) and BAS5047 (LcpD).

Mutations in *B. anthracis lcpB3* and *lcpD* caused aberrations in cell size and chain length that could be explained as discrete defects in PyrCWGP assembly. By deleting combinations of *lcp* genes from the *B. anthracis* genome, variants with single *lcp* genes were generated [43]. *B. anthracis* expressing *lcpB3* alone displayed physiological cell size, vegetative growth, spore formation, and S-layer assembly, which might implicate a direct contribution of this LCP protein to the bacterial cell cycle [116]. Strains expressing *lcpB1* or *lcpB4* displayed defects in cell size and shape, S-layer assembly, and spore formation, yet sustained vegetative growth. In contrast, the *lcpB2* strain was unable to grow unless the gene was expressed from a multicopy plasmid, and variants expressing *lcpC* or *lcpD* displayed severe defects in growth and cell shape. The *lcpB2*, *lcpC* or *lcpD* strains supported neither S-layer assembly nor spore formation.

Conclusively, it is conceivable that *B. anthracis* LCP enzymes fulfil partially overlapping functions in transferring CWGP to discrete sites within the bacterial envelope.

##### *B. anthracis* LcpB1, LcpB2, LcpB3, LcpB4, LcpC, LcpD in *S. aureus* WTA Biosynthesis

All six *B. anthracis lcp* genes were tested for their restoration capability of WTA synthesis in a *S. aureus* Δ*lcp* mutant lacking all three *lcp* genes, revealing that, with *lcpB2*, *lcpC* and *lcpD* plasmids, full complementation could be achieved. *lcpB1* was not able to restore *S. aureus* WTA synthesis at all and *lcpB3* and *lcpB4* achieved partial complementation. Thus, evidence has been obtained that LcpB2, LcpC and LcpD could transfer a WTA providing a “conventional” murein linkage unit to PGN implicating that these enzymes would display ligation activity also upon the *B. anthracis* PyrCWGP, which provides an identical murein linkage unit [115].

The open question, why *B. anthracis* employs six LCP enzymes while e.g., *B. subtilis* has evolved only three (i.e., *tagTUV*) [39] might be, at least in part, explained by the genomic organization of the CWGP biosynthesis machinery in these bacteria. In *B. subtilis*, *tagO* is part of a 50-kb genomic region that harbours virtually all genes required for WTA synthesis, including *tagTUV* [82,117].

In contrast, according to the current understanding, four different genomic loci of *B. anthracis* are linked to PyrCWGP biosynthesis, of which two encode one LCP protein each. In the *scwp1* locus, *lcpD* is encoded in close proximity to the essential *tagO* gene as well as the *gneY* gene required for PyrCWGP synthesis [118,119]. The *sps* locus (“surface polysaccharide”) encodes *lcpC* and the essential *gneZ* gene, encoding a UDP-GlcNAc-2-epimerase. The genes for the remaining four LCP enzymes—LcpB1, LcpB2, LcpB3, LcpB4—are encoded elsewhere on the genome and are not linked to genes that are known to contribute to the synthesis of PyrCWGP or a “conventional” murein linkage unit. Thus, the expanded repertoire of *B. anthracis lcp* genes could implicate that these LCP enzymes are able to attach different, not yet identified, types of *B. anthracis* CWGPs to PGN [43].

### 4.2. Firmicutes—*Order*: Lactobacillales

#### 4.2.1. *Streptococcus pneumoniae*

*Sc. pneumoniae* or pneumococcus is a major human pathogen, which typically resides in the nasopharyngeal cavity. Bacterial colonization requires interaction with host cells, for which the amount of capsule is crucial [120]. Thus, its CPS is a key virulence factor shielding *Sc. pneumoniae* from the host immune system and, thus, an important target for protective immune responses. Ninety-three capsular types have been identified serologically, and the RU unit structure of each has been defined [64]. *Sc. pneumoniae* is especially dangerous for immunocompromised people where it can cause severe diseases, including meningitis, pneumoniae or sepsis. In asymptomatic humans, *Sc. pneumoniae* resides in the nasal cavity or the sinuses, where it may cause otitis media and acute sinusitis [121].

The capsular polysaccharide serotype 2 of *Sc. pneumoniae* strain D39 (CP2) is composed of branched hexasaccharide RUs with the structure →3)[α-Glc*p*A-(1→6)-α-Glc-(1→2)]-α-L-Rha-(1→3)-α-L-Rha-(1→3)-β-L-Rha-(1→4)-β-D-Glc- (Figure 1). CP2 is directly glycosidically linked via the reducing end glucose of the RU to β-D-GlcNAc residues of PGN, without involvement of a murein linkage unit and a phosphodiester bond [122], which contrasts the usual attachment mode of CWGPs to PGN backbone sugars.

The cell wall of *Sc. pneumoniae* further contains an unusually complex WTA, which has identical RUs as the membrane-anchored lipoteichoic acid. Both show pseudo-pentasaccharide RUs containing the rare amino sugar 2-acetamido-4-amino-2,4,6-trideoxygalactose (AATGal) in addition to Glc, Rib-*P*, and two GalNAc residues that are each modified with phosphorylcholine (Figure 1) [123,124,125]. The reducing-end AATGal is proposed to be linked via a phosphodiester bond to MurNAc residues of PGN, based on the in silico identification of LCP family proteins in the *Sc. pneumoniae* genome, however, without provision of any biochemical evidence [124].

##### *Sc. pneumoniae* Cps2A, LytR, Psr, Genes and Physiological Effects

On the *Sc. pneumoniae* genome, the capsular region of CP 2 begins with the *cps2A–D* genes; all 17 capsular genes in this region are under control of the promoter upstream of *cps2A*.The first gene in the region, *cps2A*, encodes a member of the LCP protein family. Within the biosynthesis pathway of the CP2 of *Sc. pneumoniae* strain D39, the three paralogous LCP proteins Cps2A, LytR and Psr, with the latter two not localizing to the *cps* region, have been investigated.

Evidence was provided that Cps2A, LytR and Psr contribute to the maintenance of normal capsule levels and to the retention of the CP2 in the *Sc. pneumoniae* cell wall. Cps2A, LytR and Psr were found to localize at the cell membrane and accumulate at septal sites supportive of their function in cell wall maintenance. Single Δ*cps2A* and Δ*psr* mutants produced a reduced amount of capsule, while a Δ*cps2AlytR* double mutant showed greatly impaired growth and cell morphology and lost approximately half of the total capsule material into the culture supernatant [97]. Notably, inactivation of *lytR* proved to be difficult in the background of the encapsulated D39 strain and during exponential growth, LytR expression was continuously high which suggests a housekeeping function of this gene during cell division that is essential for proper septum placement [126]. According to a current data-based model, CpsA2 is responsible for the covalent attachment of CP2 to the pneumococcal cell wall, and LytR can take over this function in the absence of Cps2A.

##### Crystal Structure of *Sc. pneumoniae* Cps2A

Structural and functional studies of the Cps2A enzyme from *Sc. pneumoniae* provided the first insight into the catalytic mechanism of an enzyme from the LCP protein family. Cps2A contains a large hydrophobic tunnel that is capped with surface-exposed arginine residues that are important for catalysis [82]; serendipitously, Cps2A co-crystallizes with octaprenyl-pyrophosphate, where the isoprenyl-tail is nestled within the hydrophobic pocket with the pyrophosphate head group interacting with highly conserved arginine residues within the active site.

A ΔTM-Cps2A version of the protein comprising the accessory domain (amino acid residues 111–213) and the LCP domain (amino acid residues 214–481) has been solved at 1.69 Å-resolution. Within the LCP domain, a core containing a five-stranded β-sheet surrounded by α-helices on both faces is comprised of an overall α-β-α architecture. Between the two domains, two pairs of β-strands extend from the core site. A hydrophobic pocket is formed between the central β-sheet and α-helices 3–7, where a polyisoprenoid phosphate lipid was found. As it has been seen across the LCP superfamily, these hydrophobic side chains are conserved and arginine residues R267, R362 and R374 play a key role in the interaction between the phosphate oxygens and the formation of the pocket and are stabilized by D371 and Q378. Furthermore, magnesium ions are suggested to contribute to catalysis by implicating neutralization of the developing negative charge and are coordinated by two aspartate residues, D234 and D246 [82].

Based on the crystal structure of the soluble part of Cps2A, it was inferred that all three homologs in *Sc. pneumoniae* (Cps2A, LytR, and Psr) might attach polyprenol pyrophosphoryl-linked polymers to PGN without any further specification of the CWGP structure [97], which leaves open the possibility that these enzymes might additionally be involved in WTA attachment.

#### 4.2.2. *Streptococcus agalactiae*

*Sc. agalactiae* belongs to the GroupB *Streptococcus* (GBS); it is a common commensal organism which occurs on vaginal and rectal mucosal surfaces but is also associated with invasive infections, especially in elderly or immunocompromised patients [127]. CPS represents the main virulence factor of *Sc. agalactiae* and is a prime target in current vaccine development [128]. GBS isolates associated with human infection produce one of nine antigenically distinct CPSs [127].

The *Sc. agalactiae* CPS serotype III (CPIII) has a branched pentasaccharide structure composed of →6)-[α-NeuAc-(2→3)-Gal*p*-(1→4)]-β-Glc*p*NAc-(1→3)-β-Gal-(1→4)-α-Glc-(1→ RUs. CPIII is bound via a phosphodiester bond and an oligosaccharide linker of unknown structure composed of glucose, galactose and arabinose to GlcNAc residues of PGN [129,130] (Figure 1), representing a further variation of the PGN linkage mode.

##### *Sc. agalactiae* CpsA, Gene and Physiological Effects

The investigation of the conserved proteins CpsABCD encoded in the *Sc. agalactiae cps* operon revealed that *cpsA*, the first gene in the operon, has a regulatory function and is required for the transcription of the operon and that CpsBCD composes a phosphoregulatory system [100]. Although having no impact on *cps* transcription or the synthesis of the CPIII RU, it was suggested that these proteins are required for fine-tuning of the last steps of CPIII biosynthesis, which is balancing repeating unit polymerization and CPIII attachment to the cell wall. As a member of the LCP protein family, the 485-amino acid membrane protein CpsA is unique due to its extracellular accessory domain. It equips CpsA to specifically bind to two promoters in the *cps* locus [131]. However, a protein consisting of the accessory domain alone could not complement a Δ*cpsA* deletion strain for CPIII biosynthesis. Interestingly, even if the truncated form co-existed with the native *cpsA*, the capsule production was impaired (dominant-negative effect), suggesting involvement of CpsA in cell wall maintenance in addition to capsule expression [99]. Essential for the dominant-negative effect is a region between amino acids 210 and 245 and probably between amino acids 132 and 153 of the accessory domain of CpsA. Experiments using a fluorescent peptide showed that this effect was not due to a direct interaction of truncated CpsA with wild-type CpsA. Probably, it disturbs the mechanism associated with normal cell wall integrity and CPS synthesis [131]. Interestingly, zebrafish experiments revealed that expression of a truncated CpsA representing the accessory domain only decreases virulence stronger than the complete absence of CpsA [131].

##### *Sc. agalactiae* CpsA in CPIII Biosynthesis

In the final steps of CPIII biosynthesis, the newly synthesized pentasaccharide RU anchored to a polyisoprenoid phosphate lipid is flipped to the outer side of the bacterial membrane, where CpsH acts as the repeating unit polymerase. By analogy with other Wzy-dependent systems, polymerization occurs bottom-up. The nascent CPIII is removed from the lipid through a phosphotransferase reaction and subsequently linked to a single membrane-anchored RU. The final product is a CPS that is removed from the membrane lipid and covalently attached to GlcNAc of the PGN backbone by CpsA activity. This linkage effectively renders further polymerization impossible [100].

#### 4.2.3. *Streptococcus mutans*

*Sc. mutans* is a prototypical member of the lactic acid bacteria group. It inhabits the dental plaque biofilm community of the oral cavity and represents the most tenacious causative agent of the enamel-destructive disease dental caries [79].

As within several streptococcal species, the major cell wall antigen of *Sc. mutans* is its RhaCWGP which consists of linear rhamnose-polymers with glucose side chains (also referred to as RGP) [132,133] (Figure 1) of the RU structure →2)-[α-Glc-(1→2)]-α-Rha-(1→3)-α-Rha-(1→2)-[α-Glc-(1→2)]-α-Rha-(1→3)-α-Rha-(1→, making up approxima-tely half of the total weight of the bacterium’s cell wall [76,134].

The mature RGP contributes to acid and oxidative stress tolerance of *Sc. mutans* in the oral habitat [135] and is required for proper localization of cell division complexes in *Sc. mutans* translating in a morphogenic role for the bacterium [79]. Specifically, the serotype-specific glucose branches of the RhaCWGP from strain *Sc. mutans* serotype c were shown to act as a receptor for phage M102 [132].

##### *Sc. mutans* BrpA and Psr, Genes and Physiological Effects

*Sc. mutans* harbours two paralogues of LCP family proteins, named BrpA and Psr. Deletion of either of these did not show any impact on bacterial growth, but the mutants had major defects in acid and oxidative stress tolerance responses, defects in cell division, alterations in cell envelope morphology, and reduction in biofilm formation, probably due to missing RhaCWGP [136,137,138].

BrpA or Psr deficiency was also found to alter the expression of a number of genes, including those known to play a critical role in cell envelope biogenesis and cell division and biofilm formation, although differences exist between the two LCP proteins in the scope and effect of their gene regulation [136,137]. Specifically, in the Δ*psr* mutant, a decreased expression level of glycosyltransferase C, which is common-ly involved in biofilm formation, was found.

Importantly, one of the two LCP homologues is necessary for viability of *Sc. mutans* cells [136], which might reveal BrpA and Psr as new potential targets to develop anticaries therapeutics.

##### *Sc. mutans* BrpA and Psr in RhaCWGP Biosynthesis

Six genes (*rgpA* through *rgpF*) involved in the biosynthesis of the RhaCWGPs have been characterized by heterologous gene expression experiments in *E. coli* [139,140]. From restoration of plasmid-encoded RhaCWGP biosynthesis in an *E. coli* Δ*wecA* mutant by provision of *Sc. mutans* RgpG, it was concluded that RgpG encodes the initiation enzyme of the RhaCWGP biosynthesis in *Sc. mutans*, transferring a GlcNAc residue from UDP-GlcNAc to lipid-phosphate. According to that study, it was postulated that RgpA, RgpB, and RgpF, function in rhamnan polymerization, while RgpC and RgpD constitute an ABC transporter.

In a more recent study, allelic exchange of RgpG led to impaired cell division, reduced biofilm formation, and altered cell morphology of *Sc. mutans*. The RgpG deficient strain was used for deletion of *brpA* and *psr*. While the double mutants grew comparably to the wild-type, a *rgpG brpA psr* triple mutant showed swollen giant cells and was totally devoid of the RhaCWGP [133]. Based on this observation, the authors suggested an involvement of BrpA and Pst in attaching the RhaCWGP to the cell wall PGN, without provision of further details about the linkage.

Notably, the involvement of a WecA homologue, which is a well-known UDP-GlcNAc::lipid-*P* transferase from the LPS biosynthesis pathways [25], as an initiating glycosyltransferase in RhaCWGP biosynthesis in *Sc. mutans*, would implicate the presence of a GlcNAc residue at the reducing end of the glycopolymer. This, however, is not consistent with the current knowledge of the *Sc. mutans* RhaCWGP RU structure, leaving the option of this sugar serving as a potential linker of the capsule to the PGN.

#### 4.2.4. *Lactococcus lactis*

*Lc. lactis* is a Gram-positive bacterium widely used in dairy fermentations where it metabolizes sugars and converts these to lactic acid. In humans, lactic acid bacteria are naturally present in the gut, and, due to their GRAS (generally regarded as safe), they are considered as a convenient delivery vector for biological molecules for antiinfective and anti-allergic therapies in the gastro-intestinal tract [141,142].

*Lc. lactis* strains are covered by a RhaCWGP or sugar-phosphate polysaccharide pellicle (PSP), which is likely linked to the cell wall PGN via a “conventional” murein linkage unit as evident from the involvement of the TagO enzyme in its biosynthesis. The PSP protects the bacteria from phagocytosis in vitro and acts as the receptor for members of various lactococcal phage groups, allowing their adsorption through specific recognition events [77,143]. In strain MG1363, the anionic PSP is composed of hexasaccharide-phosphate repeats containing Glc, Gal*f*, GlcNAc, Rha and a Glc-*P* residue at the reducing-end of each repeat [77,144] (Figure 1).

In addition, the bacterium produces a neutral polyrhamnan, which is composed of linear →2)-α-L-Rha-(1→2)-α-L-Rha-(1→3)-α-L-Rha-(1→ trisaccharide repeating units [145] (Figure 1). The polyrhamnan is located underneath the surface-exposed PSP and is trapped inside the PGN as evident from a high-resolution magic angle spinning (HR-MAS) NMR analysis of an PSP-deficient *Lc. lactis* strain.

##### *Lc. lactis* LcpA and LcpB, Genes and Physiological Implications

*Lc. lactis* strains possess a large chromosomal *cwps* gene cluster, comprising a highly conserved and a variable region, with the former region involved in polyrhamnan and the latter involved in PSP biosynthesis. Thus, except for the gene *tagO* encoding the initiating glycosyltransferase, the genetic determinants of polyrhamnan biosynthesis appear to be within the same genetic locus that encodes the PSP biosynthetic machinery.

*Lc. lactis* MG1363 harbours two functional *lcp* paralogs—*lcpA* and *lcpB*–which are located outside the *cwps* gene cluster and have a monocistronic organization. Of these, only the *lcpB* gene could be successfully deleted, suggesting an essential role for LcpA in the growth and/or survival of *Lc. lactis*. An *lcpA* mutant leading to reduced expression of *lcpA* was shown to severely affect the cell wall structure. In *lcpA* mutant cells, in contrast to wild-type cells, polyrhamnan was detected by HR-MAS NMR and was, unlike the situation in the wild-type, flexibly located at the surface. This suggested that LcpA participates in the attachment of polyrhamnan to PGN, but, possibly also in attachment of the PSP, since only its absence would allow the detection of the underlaying polyrhamnan.

The current model of polyrhamnan biosynthesis follows an ABC transporter-dependent pathway, involving the production of the lipid-pyrophosphate-linked murein linkage unit, subsequently acting individual rhamnosyltransferases, and addition of a terminal sugar terminating the synthesis of the chain followed by export and ligation to PGN involving LcpA [145].

#### 4.2.5. *Lactobacillus plantarum*

The human gut microbiota contains an abundance of symbiotic lactobacilli, amongst which *Lb. plantarum* is one of the most predominant species. The organism is a key player among probiotic microorganisms and known for its high metabolic versatility [146,147].

*Lb. plantarum* strains possess 3,4-α-D-diglucosyl-2-Rbo-*P* and 3,4-α-D-diglucosyl-1-Rbo-*P* WTAs, respectively (Figure 1). The unique structure of WTA in *Lb. plantarum* results from the modification of the Rbo-*P* main chain with multiple glucose residues [148]. Of note, this WTA structure contains two monomers differing in the position of the phosphoric acid residue.

##### *Lb. plantarum* FlmA, FlmB and FlmC, Genes and Physiological Effects

In the *Lb. plantarum* genome recently, three genes–*flmA*, *flmB* and *flmC*—encoding proteins to which regulatory and cell wall-related transcriptional attenuator functions were attributed, were identified. The FlmA, FlmB and FlmC proteins all contain highly conserved C-terminal regions which appear closely related to the LCP domain [147,149] and an N-terminal TM anchor domain. By generating Δ*flm* deletion mutants, it has been shown that FlmC contributes to biofilm development and that lack of this protein results in increased autolytic activity phenotypes, whereas deletion of either of the remaining genes did not result in any significant defects [147,149].

##### Structural Model of *Lb. plantarum* FlmC

A ΔTM-FlmC (amino acid residues 81-335) structural model could be obtained based on the crystal structure of ΔTM-Cps2A from *Sc. pneumoniae*, exhibiting a typical topology of the LCP domain where conserved hydrophobic amino acid residues are centred in the inside and polar residues on the outside [147]. A hydrophobic pocket consistent with the *Sc. pneumoniae* Cps2A protein between the central β-sheet and helices 3–7 was presented in the model. The central sheet harbours six-strands surrounded on both faces by 5 α-helices [147].

The modelled structure strongly suggests that FlmC acts as a phosphotransferase, like Cps2A, as evident from the presence of similar conserved arginine residues that stabilize the binding of a lipid molecule in concert with a magnesium ion, which is fundamental for the phosphatase activity of the class of LCP enzymes [82,147].

Overall, the present data suggest that FlmC is involved in cell envelope biogenesis of *Lb. plantarum*; whether it directly affects the ligation of the bacterium’s WTA to PGN remains to be investigated.

#### 4.2.6. *Enterococcus hirae*

Commensal enterococci such as *E. hirae* are found in the normal human faecal flora; they are of interest due to their emerging pathogenicity in hospital infections which relates to biofilm formation and antibiotic resistances. Enterococci are intrinsically resistant to β-lactams due to the expression of a penicillin binding protein (PBP) displaying a low affinity for these antibiotics [150]. As with most bacteria, enterococci are dependent on their cell envelope for growth. *Enterococcus* species show WTA structures of different complexity which have not been elucidated up to now [151].

##### *E. hirae* LcpA, LcpB and LcpC

The *E. hirae* genome codes for three predicted LCP enzymes named LcpA (formerly Psr protein EHR_11445), LcpB (formerly EHR_11995) and LcpC (formerly EHR_14365), respectively. The three LCP proteins from *E. hirae* have a common topology according to in silico analysis using the TMHMM program, which shows an N-terminal cytoplasmic tail of different length depending on the protein, (six amino acids for LcpA, 103 amino acids for LcpB and six amino acids for LcpC), followed by a TM α-helix of approximately 20 amino acids (18, 23 and 20 amino acids respectively), and an LCP C-terminal domain located in the extracellular space.

##### *E. hirae* LcpA Function

Of the three *E. hirae* LCP proteins, only LcpA has been investigated. LcpA is a 293-amino acid Psr orthologue possessing a conserved 150 amino-acid LCP domain. The *lcpA* gene is located in an operon between the *ftsW* gene, coding for a SEDS (Shape, Elongation, Division and Sporulation) protein involved in lipid II export, and the *pbp5* gene, coding for a low-affinity PBP involved in PGN synthesis [20]. However, the operon is not flanked by any cluster of known CWGP biosynthetic genes.

Initially, LcpA was related to β-lactam resistance of *E. hirae* and proposed to be a repressor of penicillin-binding protein 5 (PBP5) synthesis because of a deletion found in the *lcpA* gene of the *E. hirae* strain R40, which overproduced PBP5 [152]. In subsequent studies, LcpA was found to be a membrane protein which binds *E. hirae* lysine-type PGN and localizes at the septation sites together with the low-affinity PBP5, which is involved in the late steps of PGN biosynthesis [151].

The interaction of recombinant *E. hirae* LcpA with *E. hirae* cell walls was investigated by pull-down experiments [151]. Incubation of purified *E. hirae* PGN with or without CWGP with LcpA indicated that LcpA binds enterococcal PGN regardless of the presence of WTA. Thus, it seems plausible that this LCP protein plays a role related to the cell wall metabolism, probably acting as a phosphotransferase catalysing the attachment of RhaCWGPs to the PGN of *E. hirae* [151]. This assumption is consistent with a previous finding in the *E. hirae* R40 mutant possessing a truncated *lcpA* gene which showed a decrease of the rhamnose content in its cell walls by 50%, which was not related to the overproduction of PBP5 nor to other changes in the PGN structure [153].

### 4.3. Actinobacteria—*Order*: Actinomycetales

#### 4.3.1. *Mycobacterium tuberculosis*

*M. tuberculosis* is the causative agent of tuberculosis infecting the lungs and causing about 1.5 million deaths per year [154]. What makes this organism so strong is its unique, low permeable AG-containing cell wall (Figure 1) that provides a high resistance towards antibiotics [155].

##### *M. tuberculosis* Rv0822c, CpsA1, CpsA2 and Rv3840 Function

Four genes encoding LCP proteins are annotated in the *M. tuberculosis* H37Rv genome, namely Rv0822c, Rv3267 (*cpsA1/lcp1*), Rv3484 (*cpsA/cpsA2*), and Rv3840 [156].

To investigate an association of the *M. tuberculosis* LCP proteins with the ligation of AG to PGN, the predicted enzymes devoid of the TM domain were produced recombinantly in *E. coli* and assayed in vitro. Functional proof was obtained for CpsA1 and CpsA2, which, under the chosen experimental conditions, showed pyrophosphatase activity on the generic substrate geranyl pyrophosphate in dependence on magnesium ions consistent with other LCP enzymes [65]. Notably, while individual *cpsA1* and *cpsA2* knock-outs of *M. tuberculosis* were readily obtainable, the combined inactivation of both genes appeared to be lethal.

##### *M. tuberculosis* CpsA1

*CpsA1* maps to the AG biosynthetic gene cluster where it is located immediately upstream of two genes involved in linker biosynthesis. *M. tuberculosis* CpsA1 was suggested to be the predominant enzyme responsible for the covalent attachment of AG to PGN. However, in a Δ*cpsA1* deletion mutant, no major effects were seen, probably due to functional compensation of the paralogs [65,155].

CpsA1/Lcp1 was further described to be essential for *M. tuberculosis* and its activity was verified in a cell-free radiolabelling assay with ^14^C-radiolabeled AG and nascent PGN [155]. To further evaluate the specificity of *M. tuberculosis* CpsA1, three potential AG binding substrates—L-Rha-α(1¡3)-D-GlcNAc-*O*-C_8_ (compound 1), Gal*f*_2_-Rha-GlcNAc-*O*-C_8_ (compound 2) and Gal*f*_3_-Rha-GlcNAc-*O*-C_8_ (compound 3)—were tested using intrinsic tryptophan fluorescence of CpsA1, with compound 2 showing the highest affinity with 5.13 µM [155].

Although the envelope composition was not drastically changed in the single ligase mutants, the Δ*cpsA1* mutant showed increased susceptibility to a range of antibiotics such as penicillins, vancomycin, and CPZEN-45, suggesting changes in cell wall permeability [65].

##### *M. tuberculosis* CpsA2

Although no analytically detectable difference was observed, the Δ*cpsA2* deletion mutant in the *M. tuberculosis* strain H37Rv showed a changed phenotype in an in vivo mouse model, where the mutant was not able to grow, survive and infect. Furthermore, this strain displayed increased resistance to meropenem/clavulanate and rifampicin, which could not be compensated by *cpsA1* [157]. Meropenem belongs to the carbapenem class of β-lactam antibiotics being poor substrates for BlaC, a protein encoded in the genome of *M. tuberculosis* which hydrolyses β-lactam antibiotics [158]. Rifampicin did not target the cell wall but the authors argued that the permeability had changed and, therefore, the drug did not efficiently get into the cytoplasm as supported by measuring ethidium bromide uptake and efflux [66,157]. However, Grzegorzewicz et al. could not determine increased resistance against rifampicin. According to Malm et al., this could be due to differences in the experimental set-up or resulting from a secondary effect and not from *cpsA2* deletion [65,157].

##### Additional *M. tuberculosis* Proteins Related to LCP Enzyme Function

Recently, mutants of the “cell envelope integrity” gene (*cei*; Rv2700) of *M. tuberculosis* and its structural homolog VirR (Rv0431), showed a decreased growth rate at low densities, increased susceptibility towards antibiotics (vancomycin, meropenem and rifampicin), increased sensitivity to nitric oxide (NO), and increased cell envelope permeability. In addition, a Δ*cei* mutant did not lethally infect mice, where one factor that influences virulence is the growth control by NO after infection [66,159].

Importantly, these newly detected gene products have one predicted TM domain and a LytR_C domain, which are features found in LCP enzymes commonly referred to as LytR_C-only proteins. Due to very similar phenotypes of the knockout mutants of *cei* and *virR* to common LCP knockout strains, a relation to and common participation in the same pathway, namely AG ligation to PGN, was hypothesized [66].

#### 4.3.2. *Mycobacterium marinum*

*M. marinum* is a slow-growing, acid-fast bacterium in the category of non-tuberculous mycobacteria which most commonly cause skin and soft tissue infections in patients, particularly those with aquatic exposure [160]. The bacterium possesses an AG as is characteristic of mycobacteria.

##### *M. marinum* MMAR_4858, MMAR_1274, MMAR_4966 (CpsA), and MMAR_5392

*M. marinum* (MMAR) harbours orthologues of all LCP proteins found in *M. tuberculosis* complex (Mtbc) strains—namely MMAR_4858, MMAR_1274, MMAR_4966 (CpsA), and MMAR_5392 in MM for Rv0822c, Rv3267, Rv3484, and Rv3840 for Mtbc [65,157].

A strain defective in the CpsA2 (Rv3484) orthologue in *M. marinum* (CpsA) showed impaired growth in vitro in contrast to CpsA deficient strains of *M. tuberculosis* [65,157]. Furthermore, the *M. marinum* Δ*cpsA* mutant revealed alterations in colony morphology and cell surface properties, increased susceptibility to antibiotics (erythromycin, vancomycin and penicillin), and a change in cell wall permeability for hydrophobic components. Traditional analytics of the cell wall composition indicated an imbalance in the AG/PGN ratio indicative of a role of the MMAR_4966 enzyme in AG transfer to PGN. Finally, the transposon mutant was severely attenuated in the zebrafish model and growth impaired in the murine macrophage cell line RAW 264.7 [161].

#### 4.3.3. *Corynebacterium glutamicum*

*C. glutamicum* is a well-established model species for cell wall-related studies in the *Corynebacteriales* because it shares the complex cell envelope organization with its pathogenic relatives, such as *M. tuberculosis* [162]. It contains a mycolyl-AG-PGN complex (compare with Figure 1) in addition to lipo(arabino)mannan in its cell wall. Furthermore, *C. glutamicum* is one of the main species used in the biotechnological industry, especially for the production of amino acids [163].

##### *C. glutamicum* LcpA and LcpB

As described for *Sc. pneumoniae* and *E. hirae*, *C. glutamicum*’s two LCP proteins, LcpA and LcpB, localize where nascent cell wall biosynthesis happens. Interestingly, of the two *C. glutamicum* LCP proteins, a deletion was only feasible for LcpB, but did not lead to any detectable changes in the cell wall as compared to the wild-type strain.

LcpA could be conditionally silenced, which influenced bacterial growth, the ratio of cell wall components, and morphology. Compared to the wild-type cell wall, the cell wall of the *C. glutamicum* Δ*lcpA* mutant contained significantly less mycolic acids and a reduced amount of AG. In particular, rhamnose, a specific sugar component of the linker that connects AG and PGN was decreased (compare with Figure 1).

Characteristic of LcpA is the presence of an LCP domain and a LytR_C domain, which is frequently found in actinobacteria. In complementation studies, the importance of the conserved arginine and aspartate residues in the LCP domain as well as the general importance of the LytR_C domain was shown [164]. LcpA was shown to oligomerize into dimers or tetramers, wherefore the LytR_C domain might be responsible supportive of the LytR_C domain catalysing its own reaction [164].

#### 4.3.4. *Streptomyces coelicolor*

*Sm. coelicolor* is the genetically best-known representative among the soil colonized, filamentous Gram-positive bacteria of the *Streptomycetes* genus [165], which play an important role in producing natural antibiotics.

In contrast to most other bacteria, which divide by binary fission *Sm. coelicolor* A3(2) develops a mycelial lifestyle by apical tip extension, which requires a dedicated mode of PGN incorporation [166].

*Sm. coelicolor* A3(2) encodes several homologues of Tag proteins [167] directing WTA synthesis in *B. subtilis*, although the major glycopolymer of Sm. coelicolor is teichulosonic acid. This teichulosonic acid is a phosphate-free polymer of up to seven RUs composed of galactose and the neuraminic acid-related 2-keto-3-deoxy-D-*glycero*-D-*galacto*-nononic acid (Kdn), often substituted with GlcNAc or a methyl group. As a minor component, a polydiglycosylphosphate CWGP (referred to as PDP) consisting of →6)-α-Gal*p*-(1→6)-α-Glc*p*NAc-*P*-(1→- RUs is present in *Sm. coelicolor* cell walls [168] (Figure 1).

##### *Sm. coelicolor* PdtA and Ten Other LCP Proteins

The genome of *Sm. coelicolor* harbours 11 LCP proteins, with seven genes (SCO3042-SCO3048) clustered like the *B. subtilis* TagT, TagU, or TagV-like phosphotransferae genes [169].

PdtA (SCO2578), a TagV-like phosphotransferase, is suggested to be co-transcribed with a nicotinate-nucleotide adenylytransferase gene SCO2579 and is in close proximity to other presumed CWGP-linked genes [167,169]. It was identified in a screen of interaction partners of several *Streptomyces* spore wall-synthesizing complex (SSSC) proteins possibly involved in sporulation [170]. PdtA inactivation resulted in irregular spore chains; more precisely, the placement of sporulation septa was affected and heterogeneity in spore sizes was displayed [169].

The other 10 LCP homologs did not show any severe phenotype effects and were not able to substitute for PdtA, upon whose deletion, a 48% reduction of spore wall glycopolymer content was determined, comparable to the situation in other LCP-containing organisms [82,169].

Interestingly, of the two types of *Sm. coelicolor* CWGPs, only PDP was affected in the spore envelope by the lack of *pdtA* and resulted in a severe phenotype, including imprecise sporulation septa placement and spore viability decrease by one-third. The remaining spores had increased sensitivity to osmotic stress and lysozyme. Furthermore, the Δ*pdtA* mutant showed impaired vegetative tip growth and, interestingly, also high sensitivity to rifampicin, which is known to cross the bacterial cell wall targeting the RNA polymerase and to have no effect on cell envelope synthesis [169,171]. Possibly, PDP acts as a barrier to block large-sized antibiotics, such as rifampicin [169].

It was speculated that PDP is anchored to the hyphal tip by PdtA resulting in apical tip growth, which aids as a framework for PGN synthesis, particularly under stress conditions [169]. The crucial role of PdtA under stress conditions becomes even clearer, as under high-salt conditions, only 4% of normal biomass was produced and hyphae showed aberrant morphology. Conclusively, of the 11 LCP protein homologs found in *Sm. coelicolor*, PdtA is the only protein involved in PDP synthesis and plays a key role for the life cycle of the organism [169].

#### 4.3.5. *Actinomyces oris*

The actinobacterium *A. oris* is a colonizer of the oral cavity where it plays a specific role in the formation of supragingival plaque [172]. *A*. *oris* is dependent on the activity of its SrtA enzyme, which is conditionally dependent on glycosylation of the GspA surface protein by the activity of an LCP enzyme [173].

##### *A. oris* LcpA Participates in Protein Glycosylation

The LCP homolog LcpA of *A. oris* provide a so far unique example of an LCP enzyme that is involved in a protein glycosylation process, i.e., the transfer of a saccharide moiety to an amino acid acceptor sequence [174] instead of a PGN backbone sugar. LcpA is genetically linked to GspA, a glycoprotein that is attached to *A. oris* PGN by the house-keeping sortase SrtA, which, in turn, recognizes the cell wall sorting signal of the glycoprotein [175]. Deletion of either the *gspA* or *lcpA* gene resulted in rescue effects of *srtA* depletion, leading to the suggestion that excessive aggregation of GspA proteins causes stress leading to cell death, whereas the absence of GspA in the presence of SrtA and LcpA is not lethal [176]. The neighbouring genes *gspA* and *lcpA* in *A. oris* implicate that their glycoprotein products are linked to each other and that the high glycosylation level of GspA involves LcpA [174]. LcpA glycosylates GspA along an unknown pathway, prior translocation across the cytoplasmic membrane and final cell wall anchoring by the sortase SrtA [176]. However, the exact nature and composition of the GspA glycans remain to be determined.

##### *A. oris* LcpB, LcpC and LcpD

Interestingly, *A. oris* MG1 encodes three other LCP domain-containing proteins, LcpB (ana_0299), a homolog of the TagF glycosyl/glycerophosphate transferase from *Staphylococcus epidermidis* [177], LcpC (ana_1577) and LcpD (ana_1578), which are found in the same transcriptional unit [174]. Mutant strains were generated by deletion of *lcpB* and *lcpD* as well as a triple mutant *lcp*Δ*3*, devoid of *lcpA*, *lcpB* and *lcpD*, to analyse LcpA-mediated glycosylation. The single mutants did not show any negative effect regarding the formation of high-molecular-mass GspA species with attached glycans, whereas the triple mutant did, supporting that LcpA is necessary and sufficient for the production of glycosylated GspA.

Together with the obtained crystal structure (see below), this is the first experimental evidence of the glycosylation capability of LcpA which occurs prior to GspA glycoprotein transfer to PGN [174]. The data suggest that LcpA is the only enzyme involved in GspA glycosylation in *A. oris*, but it has to be mentioned that a Δ*lcpC* deletion mutant could not be generated and possibly may also modify GspA [174].

##### Crystal Structure or *A. oris* LcpA

A notable structural variant of LCP enzymes is that of *A. oris* LcpA, which possesses unique structural features around the active site presumably associated with binding target proteins rather than PGN for glycosylation.

The molecular structure of the extracellular LcpA domain has been resolved at 2.5-Å (eLcpA, residues 78–360). The core of the protein is formed by seven-stranded antiparallel β-sheets flanked with eight α-helices on both sites, forming ~23-Å hydrophobic tunnel [174]. Its presence is consistent with other members of the LCP protein family, leading into the active sites of LcpA that possibly bind a lipid-linked glycan substrate [82,174]. Conserved arginine residues R128, R149 and R266 were identified clustering within a pocket exposed on the surface, which indicates mediated phosphotransfer of glycopolymers as known in the LCP family [82,97,147,174]. By generating alanine substitution mutants of these arginine residues, it could be determined that the R149 and R266 residues are essential for LcpA glycosylation activity on GspA. Interestingly, LcpA links the C-terminus to α-helices between C179 and C365 formed by a presumably stabilizing disulphide bond that is also found in other actinobacterial LCP proteins [174]. Alanine substitution mutants of either one or both Cys residues in LcpA suggested that the disulphide bond is essential for protein stability as evident from a defect of mutant LcpA membrane expression as well as for full enzymatic activity [174].

Based on the demonstrated in vitro pyrophosphate activity of TagT [82], an in vitro assay of eLcpA over quantification of released inorganic phosphate in concert with a diphosphate mimic substrate showed that LcpA displays pyrophosphate activity and corroborated the necessity of the disulfide bond for catalysis [174]. This distinct feature seems to be common in actinobacterial LCP enzymes and has not been found in other LCP enzymes studied to date [174]. Overall, the structure of eLcpA seems closely related to the TagT enzyme from *B. subtilis* [97,174]. However, unlike TagT, LcpA is not capable of attaching CWGPs to PGN but to GspA instead [174].

### 4.4. Cyanobacteria—*Order:* Nostocales

#### 4.4.1. *Anabena* sp.

The filamentous cyanobacterium *Anabaena* sp. strain PCC 7120 is a commonly used model organism to study cyanobacterial nitrogen fixation and cell differentiation [178]. It is capable of fixing carbon dioxide by oxygenic photosynthesis or of fixing molecular nitrogen when a combined nitrogen source such as ammonium or nitrate is not available; the bacterium segregates these two incompatible processes by multicellular development by differentiating 5–10% of vegetative cells into so-called heterocysts [179].

A polysaccharide layer is placed over the *Anabena* sp. proheterocyst during maturation followed by a glycolipid layer between the polysaccharide layer and the outer membrane aimed at diminishing oxygen entry into the cell [179,180,181].

##### *Anabena* sp. ConR

The gene all0817 named *conR* (constriction regulator) is predicted to contain an LCP domain. While *conR* was initially predicted to be a transcriptional regulator [182], its deletion caused diazotrophic growth and heterocyst differentiation defects [179]. Although the polysaccharide and glycolipid envelope layers were present in the mutant, the polar junctions connecting heterocysts to vegetative cells were incomplete or widely open, which was hypothesized to allow oxygen to enter the heterocysts and inactivate nitrogenase [182].

Furthermore, the expression of *conR* was upregulated after nitrogen step-down in differentiating heterocysts and vegetative cells. In nitrate-containing media, filaments of the Δ*conR* mutant strain also showed aberrant septum formation of vegetative cells and defects in cell separation. However, after nitrogen step-down, the defective vegetative cells seemed less severe compared to filaments in nitrate-containing media [179]. It was suggested that these phenotypic growth defects do not simply evolve from defective nitrogen fixation but rather from a disrupted delivery of fixed nitrogen from heterocysts to their neighbouring vegetative cells via non-specific intracellular channels. The defective septum formation in the mutant could possibly result in deformation of these channels at the junction between vegetative cells and heterocysts, leading to aberrant metabolite exchange [179].

Conclusively, the putative LCP protein ConR in *Anabena* sp. is developmentally regulated and is essential for diazotrophic growth and heterocyst morphogenesis; specifically, it was found to be associated with septum formation and cell wall maintenance.

## 5. Conclusions

The LytR-CpsA-Psr (LCP) phosphotransferases are present in virtually all Gram-positive bacteria, which characteristically contain a high proportion of PGN-attached CWGPs (Table 1). Accumulated structural and biochemical data on several LCP proteins from various bacterial species provide strong evidence that this family of proteins carries out the key step of attaching CWGPs to the cell wall PGN. Thus, in-depth investigations of LCP proteins may not only aid our understanding of Gram-positive cell wall assembly but may also reveal important aspects of a novel antibiotic target.

It is noteworthy that LCP-encoding genes often but not necessarily localize in close proximity to the gene cluster encoding the CWGP for which they exert their catalytic effects [89] and that, in most bacteria, LCP proteins are present in multiple copies expressing in part functional redundancy.

Mechanistically, LCP proteins typically hydrolyse the pyrophosphate linkage between the lipid-carrier and the reducing-end sugar of the CWGP (i.e., they hydrolyse the linkage created by an NDP-sugar::lipid phosphate transferase)—in most but not all cases, this sugar is part of a dedicated murein linkage unit—and attach the CWGP to either MurNAc or GlcNAc residues of the PGN backbone via a phosphate ester linkage [82,89,97]. Recent exceptions from this picture of LCP proteins come from *Sc. pneumoniae*, where CP2 is directly glycosidically attached to GlcNAc residues of PGN by LCP activity of CpsA2 [122] and from *A. oris*, where the LCP domain-containing LcpA protein is involved in protein glycosylation [174].

The extracellular, soluble domains of several homologous LCP proteins in, e.g., *Sc. pneumoniae* and *B. subtilis*, have been crystallized and were proposed to be responsible for hydrolysis of the pyrophosphate linkage between the CWGPs and the membrane lipid anchor and subsequent attachment of CPS to the PGN. Further structural characterization of LCP enzymes is required to investigate PGN binding and to clarify the structural relationship between the donor lipid headgroup and the enzyme [106].

## Figures and Tables

**Table 1:** Summary of LCP proteins discussed in this review ^1^.

Bacterium	LCP Name	Accession No.	Transferred CWGP	Linker	Acceptor	In Vitro Assay		References
						Substrate	Acceptor	
*A. oris*	LcpA	AXE84_RS08820	Unknown saccharide	n.d.	GspA protein			[174]
*Anabena* sp.	ConR	all0187	Unknown PS	n.d.	n.d.			[179]
*B. anthracis*	LcpB1 LcpB2 LcpB3 LcpB4 LcpC LcpD	BAS1830, BAS0572, BAS0746, BAS3381, BAS5115, BAS5047	PyrCWGP	β-D-Man*p*NAc-(1→4)-α-D-Glc*p*NAc-(1→*P*	MurNAc/PGN			[43]
*B. subtilis*	TagT TagU TagV	BSU_35840, BSU_35650, BSU_35520	WTA	β-D-Man*p*NAc-(1→4)-α-D-Glc*p*NAc-(1→*P*	MurNAc/PGN	Lipid β	mature PGN	[109]
*C. glutamicum*	LcpA LcpB	Cg0847/NCgl0708 Cg3210/NCgl2802	AG	α-L-Rha*p*-(1→3)-α-D-Glc*p*NAc-(1→*P*	MurNAc/PGN			[164]
*E. hirae*	LcpA LcpB LcpC	EHR_11445 EHR_11995 EHR_14364	Unknown WTA	n.d.	PGN			[151]
*Lb. plantarum*	FlmA FlmB FlmC	LP_RS02565 LP_RS01195 LP_RS04280	WTA	β-D-Man*p*NAc-(1→4)-α-D-Glc*p*NAc-(1→*P*	MurNAc/PGN			[147,149]
*Lc. lactis*	LcpA	llnz_02385	RhaCWGP polyrhamnan	β-D-Man*p*NAc-(1→4)-α-D-Glc*p*NAc-(1→*P* β-D-Man*p*NAc-(1→4)-α-D-Glc*p*NAc-(1→*P*	MurNAc/PGN			[145]
*M. marinum*	CpsA	MMAR_4966 MMAR_4858 MMAR_1274 MMAR_5392X	AG	α-L-Rha*p*-(1→3)-α-D-Glc*p*NAc-(1→*P*	MurNAc/PGN			[161]
*M. tuberculosis*	Rv0822c CpsA1/Lcp1 CpsA/CpsA2 Rv3840	Rv0822c Rv3267 Rv3484 Rv3840	AG	α-L-Rha*p*-(1→3)-α-D-Glc*p*NAc-(1→*P*	MurNAc/PGN	Gal*f*_2_-Rha-GlcNAc-*O*-C_8_ Geranyl-PP	Un-crosslinked PGN	[65,155]
*S. aureus*	LcpA LcpB LcpC	MsrR SA0908 SA2103	WTA CP5	β-D-Man*p*NAc-(1→4)-α-D-Glc*p*NAc-(1→*P* RU β-D-Fuc*p*NAc-(1→*P*	MurNAc/PGN	C_30_-MLU Lipid I_cap_	Un-crosslinked PGN Lipid II _PGN_	[90,98]
*Sc. agalactiae*	CpsA	SAG_RS08600	CPIII	OS-(1→*P*	GlcNAc/PGN			[131]
*Sc. mutans*	BrpA Psr	SMU_RS01995 SMU_787	RhaCWGP	Glc*p*NAc-(1→*P*	PGN			[136,137,138]
*Sc. pneumoniae*	Cps2A LytR Psr	SPD_0315 SPD_1741 SPD_1202	CP2 WTA	β-D-Glc*p*-(1→ RU AATGal*p*	GlcNAc/PGN			[97,126]
*Sm. coelicolor*	PdtA	SCO2578 SCO3042 SCO3043 SCO3044 SCO3045 SCO3046 SCO3047 SCO3048 SCO4755 SCO5358 SCO6020	PDP	RU α-D-Glc*p*NAc-(1→*P*	MurNAc/PGN			[183]

^1^ Matching compounds in a bacterium are shown in the same color. n.d., not determined; RU, designates that the reducing-end sugar of the repeating unit is linked to PGN; MLU, “conventional” murein linkage unit with the structure β-D-Man*p*NAc-(1→4)-α-D-Glc*p*NAc.

## Abbreviations

4,6Pyr	4,6-ketal-pyruvate
AATGal*p*	α-D-2-*N*-acetylamido-4-amino-*galacto*-pyranose
Ac	acetyl
*A. oris*	*Actinomyces oris*
AG	arabinogalactan
Ara	arabinose
*B. anthracis*	*Bacillus anthracis*
*B. subtilis*	*Bacillus subtilis*
*C. glutamicum*	*Corynebacterium glutamicum*
C6-OH	hydroxyl group at carbon 6
CP2	capsular polysaccharide serotype 2
CP5	capsular polysaccharide serotype 5
CPS	capsular polysaccharide
CWGP	cell wall glycopolymer
*E. hirae*	*Enterococcus hirae*
*f*	furanosidic
Fuc	fucose
FucNAc	*N*-acetylfucosamine
Gal	galactose
GalNAc	*N*-acetylgalactosamine
GBS	group B *Streptococcus*
Glc	glucose
GlcNAc	*N*-acetylglucosamine
Gro-*P*	glycerolphosphate
*Lb. plantarum*	*Lactobacillus plantarum*
*Lc. lactis*	*Lactococcus lactis*
LCP protein	LytR-CpsA-Psr -glycopolymer transferase-
LPS	lipopolysaccharide
*M. marinum*	*Mycobacterium marinum*
*M. tuberculosis*	*Mycobacterium tuberculosis*
ManNAc	*N*-acetylmannosamine
ManNAcA	*N*-acetylmannosaminuronic acid
MRSA	Methicillin-resistant *Staphylococcus aureus*
MSSA	Methicillin-sensitive *Staphylococcus aureus*
MurNAc	*N*-acetylmuramic acid
NDP	nucleosidediphosphate
NeuAc	*N-*acetylneuraminic acid
*P*	phosphate
*p*	pyranosidic
PDP	polydiglycosylphosphate
PGN	peptidoglycan
R	arginine
Rbo-*P*	ribitolphosphate
RGP	rhamnose-glucose polysaccharide
Rha	rhamnose
RhaCWGP	rhamnose-containing cell wall glycopolymer
RU	repeating unit
PBP5	penicillin-binding protein 5
PyrCWGP	pyruvylated cell wall glycopolymer
*S. aureus*	*Staphylococcus aureus*
*Sc. mutans*	*Streptococcus mutans*
*Sc. pneumoniae*	*Streptococcus pneumoniae*
*Sc. agalactiae*	*Streptococcus agalactiae*
S-layer	bacterial cell surface layer
SLH	S-layer homology
*Sm. coelicolor*	*Streptomyces coelicolor*
sp.	species
undp-*P*	undecaprenylphosphate
WTA	wall teichoic acid
